# Optimized Deep Neural Network and Its Application in Fine Sowing of Crops

**DOI:** 10.1155/2022/3650702

**Published:** 2022-08-21

**Authors:** Bing Li, Jiyun Li

**Affiliations:** College of Modern Information Technology Henan Polytechnic, ZhengZhou 450018, China

## Abstract

Winter wheat is one of the most important food products. Increasing food demand and limited land resources have forced the development of agricultural production to be more refined and efficient. The most important part of agricultural production is sowing. With the promotion of precision agriculture, precision seeding has become the main component of modern agricultural seeding technology system, and the adoption of precision seeding technology is an important means of large-scale production and cost saving and efficiency enhancement. However, the current sowing technology and sowing equipment cannot meet the requirements of wheat sowing accuracy. In this context, a differential perturbation particle swarm optimization (DPPSO) algorithm is proposed by embedding differential perturbation into particle swarm optimization, which shows fast convergence speed and good global performance. After that the DPPSO is used to optimize the convolutional neural network (CNN) to build an optimized CNN (DPPSO-CNN) model and applied to the field of crops fine sowing. Finally, the experimental results show that the proposed method not only has a faster convergence rate but also achieves better wheat seeding performance. The research of this paper an effectively improves the accuracy and uniformity of wheat seeding and lay a foundation for improving wheat yield per unit area and promotes the intelligent development of agriculture in the future.

## 1. Introduction

Food security is an important strategic issue concerning China's economic development and social stability. As a country with a large population in the world, China should attach great importance to food security at all times [1, 2]. Since the beginning of the new century, the central government has successively issued no. 1 documents, which have made great achievements in agriculture and rural areas. In 2020, China's grain and other agricultural products will have a bumper harvest, and the total grain output will reach 1,339 billion Jin. At the same time, it is very difficult for farmers to feed their families only by growing grain without relying on sideline work or migrant work, and a large number of agricultural labor force has flooded into the cities, and China's food security depends on the left-behind people who struggle to make a living by growing grain, and it is increasingly unsustainable [3, 4].

China is a big agricultural country, and wheat is one of the most important grain crops in China. The population whose staple food is wheat accounts for about 1/3 of the world's total population. Therefore, ensuring high and stable wheat yield is of great significance to food security. Agricultural production is a necessary condition for the survival and development of human society, closely related to social stability and economic development, and is the most important social production activities of human beings [5]. The development of wheat industry is directly related to food safety and social stability in China. The annual consumption of wheat products accounts for about 20% of the total food consumption in China [6].

As the key link of wheat production, sowing affects the growth and development of wheat, and ultimately affects the yield of wheat [7]. In the process of wheat production, there are mechanical drill sowing, broadcast sowing, and set sowing, etc. In the actual production, due to the contradiction between rice–wheat rotation system and wheat seeding in South China, the production is mainly based on artificial broadcast sowing and extensive management, which increases the yield of wheat. Strengthening the research on new variety breeding and cultivation technology has a significant impact on the development of wheat productivity. The first is the success of wheat breeding, and the corresponding wheat breeding agronomy needs corresponding farming tools. Second, uniform plant distribution will increase the yield, which indicates the direction for the study of precision seeding in a plot. Precision sowing needs to be applied to the original seed quantity, quality, and other indicators to control, so as to complete the control of seeding quantity and quality to achieve the purpose of precision sowing [8]. Precision seeding device can complete the precise seeding process, but precision seeding is a complex organic combination, including the precise control of seeding depth and seeding position. Although the consistency of seeding depth can be achieved by seeding machine, the cost is too high. To sum up, precision sowing is the result of multiple factors, and a single analysis of seed metering device is not comprehensive. Therefore, it is necessary to combine machine-learning methods to increase the description of iodine excess in the sowing process, which is of great significance to promote precision sowing.

Compared with western developed countries, China's wheat production mode is relatively backward, mainly in the traditional way of planting, sowing, and fertilization according to artificial experience [9]. Planting closely can lead to crowding of crop seedlings and insufficient light, thus increasing the labor density. . Too little sowing will lead to inadequate land use and affect crop yield. Therefore, the realization of precision sowing and application of crops and the promotion of precision agriculture are not only of great significance to improve crop yield and reduce production costs, but also imperative. Precision agriculture is a modern agricultural production system based on modern information and space technology, which is based on remote sensing technology, geographic information system, and global positioning system to achieve precise agricultural operations [10, 11]. According to the specific conditions of each unit inside the farmland area, the soil nutrition information and the spatial status of productivity, the rational use of crop input determine the production target.

At present, the acquisition of crop growth information technology with high accuracy, high speed, high density, and low cost is still the biggest obstacle to the implementation of precision agriculture [12]. The traditional method of field sampling is to understand wheat-sowing situation, but due to the large manpower and material resources consumption of sampling and experiment, the amount of information collection and sampling cost is contradictory. Traditional precision agriculture variable implementation to obtain target data time-consuming, high cost, and time lag, cannot reflect the real-time sowing of wheat. Deep learning technology can provide timely information for agricultural production decision-making and management and provide new approaches and methods for crop growth, quality, and yield monitoring and regional management. Deep learning technology is an important means to collect physical and chemical data of ground objects and their spatio-temporal change information [13, 14]. It has been widely used, especially with the development of hyperspectral remote sensing technology. Because it can measure the main information needed for wheat seeding and fully display its growth characteristics, it can obtain more abundant information than the conventional method, so as to realize the fine monitoring of wheat seeding. To sum up, wheat fine sowing benefits the country and the people, and the development of deep learning brings convenience to the evaluation and analysis of seeding effect. On this basis, it is of great significance to analyze wheat growth and spatial variation [15].

## 2. Related Work

The water consumption of wheat from sowing to overwintering was mainly distributed in the shallow soil layer of 60 cm. The water-consuming layer moved from shallow layer to deep layer as the temperature increased from rising stage to mature stage. The water use efficiency decreased with the increase of planting density. If the sowing rate is too high or too low, the soil water storage in the early stage will be overused and the water consumption of winter wheat will be reduced throughout the growth period [16, 17]. If the amount of sowing, the number of basic seedlings in the early stage, and the total tiller number and leaf area index were large too large, then all these factors lead to the decrease of leaf area in the middle and late stage than that in the low sowing. When the amount is small, the population per unit area is insufficient, resulting in low dry matter quality. The tillering capacity and material production capacity of wheat decreased when the amount of sowing was large, and finally the grain quality decreased [18].

With the increase of sowing amount, the number of grains per spike and 1000-grain weight of wheat decreased gradually over the small sowing amount, while the number of ears increased gradually with the sowing amount, and the number of ears was the highest under the large sowing amount. Under the condition of high sowing amount, the yield did not increase but decreased slightly with increasing sowing amount. The main effect of sowing rate on yield was panicle number, followed by grain number per panicle and 1000-grain weight. Increasing sowing amount could effectively increase panicle number, but grain number per panicle weight decreased, and the positive effect of increasing panicle number was greater than the negative effect of decreasing grain number per panicle weight. Nitrogen absorption efficiency and nitrogen production efficiency increased with the increase of sowing amount. With the increase of planting density, the assimilate transport decreased before anthesis, but the accumulation of assimilate and its contribution rate to grain increased after anthesis due to the influence of soil moisture and sunlight, and finally increased protein content. Medium and low sowing rate can not only increase the yield but also significantly increase the content of starch and protein in grain, so that the grain yield and quality can be improved synchronously. Suitable medium sowing amount could increase protein content at maturity stage, and before the suitable sowing amount, protein content gradually increased with the increase of sowing amount and decreased when the suitable sowing amount exceeded [19, 20].

The appearance characteristics of granular fertilizer and wheat seed are similar, so the existing control system of granular fertilizer application amount has important reference significance to the research and development of wheat seeding amount control system. It can be seen from the current situation of foreign research that some seeding quantity control systems are still controlled by open-loop system, and even closed-loop system is controlled by indirect seeding quantity. In actual sowing operations, there is no breakthrough in the technology of accurate monitoring of large flow sowing quantity of wheat [21]. Therefore, in the process of literature research, there is no sowing quantity control system that can feedback the actual sowing quantity. Through the literature review, it can be seen that the domestic seeding quantity control system is mainly an open-loop system. If the friction between the seeding shaft and the machine and tools is large or there is an installation error, there is an error between the rotation speed of the seeding shaft and its theoretical value, and the rotation speed of the seeding shaft is not uniform within one week which will seriously affect the accuracy of seeding uniformity and seeding amount [22].

According to domestic and foreign practical experience, the advanced agricultural technology depends on the progress of agricultural production machinery. The current pattern of wheat precision sowing in China is also the most recognized by farmers [23, 24]. After that, the speed of seed wheel is controlled by the intelligent speed regulation system, so as to achieve uniform sowing of wheat seed. In this process, it is often necessary to have a fixed power source to provide power for the seed feeder, and the common power source is the ground wheel. However, due to the special properties of ground wheel drive, it has certain requirements on the size of seed besides the loss of seed and ridging, and only the wheat seeds that meet the requirements can be precisely sown. In particular, the poor stability of the power source has always limited the accuracy of seeding, so it is easy to form the instability of plant spacing, and serious shortcomings will also appear in ridging and lumps of seedlings. In the actual production process, due to the consideration of cost, the intelligent precision control system of this type of seeder is often missing, resulting in the adjustment of seeding quantity that is not accurate and cannot meet the most basic precision seeding requirements. At present, air-suction seeder is mainly oriented to large seeds, such as beans, cotton, which is generally economic crops and mainly applied to corn in the field of food crops [25, 26]. However, because wheat belongs to small seeds, air-suction seeder is not suitable for large seeds, and the existing small-seed seeder is mainly used for rapeseed, pepper, and other cash crops, so the type and number of air-suction seeder suitable for wheat are not very common.

The research on precision sowing in agricultural developed countries abroad is earlier, which can be traced back to the middle of the last century. Precision sowing can not only save seeds but also improve the quality of sowing, thus playing an important role in improving crop yield. Therefore, precision sowing has become the development trend of the sowing industry once it came into being. The same type of precision planter is divided into different series to meet the requirements of different rows, spacing, and traction power. For example, the NC model of MONOSEM precision planter in the United States can realize 4–12 rows of simultaneous seeding. The spacing between rows is 35–80 cm and can realize the simultaneous sowing of 6–24 rows. The line spacing is 45–50 cm, and different types of fine seeding machine can meet the requirements of different ground conditions, soil conditions, and crops by replacing different structures or specifications of the working parts. Precision seeding is divided into mechanical type and pneumatic type. Compared with mechanical type, pneumatic type seed metering device pushes the seeds forward by the force of airflow [27, 28]. It has the advantages of fast seed dividing speed and noninjury and can realize the sowing of different seeds through the replacement of the seed metering plate, with high versatility. In the 1980s, the agricultural developed countries represented by the United States focused their attention on the research of pneumatic precision seeder, and it has been widely used. With the development of research, many modern technologies have been applied to precision seeding machines. In the 1990s, Japan developed a seeder that could be controlled by solenoid valve and developed an electronically controlled precision seeder. The precision seeder has high precision and can control the amount of seeding in real time, which greatly improves the sowing efficiency. This study not only broadens the research idea for the researchers of wheat fine seeding but also has great significance for the development of wheat industry, since the CNN model proposed in this paper is a typically deep learning model, and it can effectively deal with big data situations. The main contributions of this paper are the following:

1. DPPSO-CNN is applied in the field of fine sowing of crops for the first time in this paper.
2. The method in this paper not only has solid theoretical foundation but also has broad application prospect.

## 3. Optimized CNN for Fine Sowing of Crops

### 3.1. Deep CNN Model Introduction

**Figure 1:**
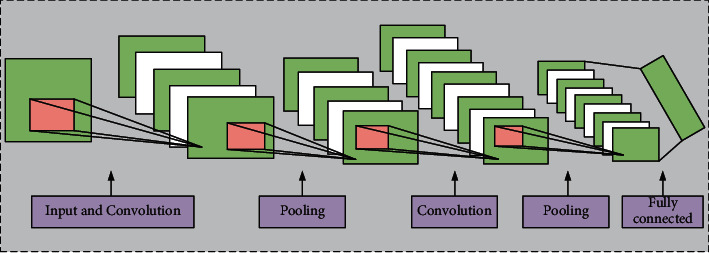
The typical schematic diagram of CNN.

In recent years, CNN model is often used to solve complex image recognition problems [29, 30]. Based on the traditional full-connection layer neural network, CNN adds convolution layer and pooling layer to form the deep CNN model, which is shown in Figure 1. As Figure 1 only shows the schematic diagram of CNN algorithm in this paper, it is impossible to know how many convolutional layers and pooling layers there are. In the algorithm of this paper, we set two layers of pooling layer and two layers of convolution layer, respectively.

The function of the convolution layer lies in the extraction of image features. The essence of the convolution kernel is a filter matrix, which can produce many different effects on the original image. The calculation process of convolution is as shown in equation (1):

(1) $$\begin{array}{c} {\text{CONV}}_{\left(ij\right)}=\sum_{i}^{m-1}\sum_{j}^{n-1}u_{ij}\times w+b\;\left(i=1,2,\ldots ,m-1;j=1,2,\ldots ,n-1\right), \end{array}$$

where, *u*_*ij*_ is the input image, *m* and *n* are the sizes of the input image, *w* is the size of the convolution kernel, and *b* is the bias constant of the convolution kernel. CONV(*ij*) is the characteristic graph output after convolution operation.

CNN adds an activation function layer to the network and analyzes the model better by adopting the feature mapping method of nonlinear function. Then, the mathematical expression of common activation function is introduced one by one. The mathematical expression of sigmoid function is

(2) $$\begin{array}{c} f\left(x\right)=\frac{1}{1+e^{-x}}. \end{array}$$

Since formula (1) is an almost function, the value range of its independent variables is the whole real number, and the range of its dependent variables is [−1,1]. The mathematical expression of tanh function is

(3) $$\begin{array}{c} f\left(x\right)=\frac{e^{x}-e^{-x}}{e^{x}+e^{-x}}. \end{array}$$

The mathematical expression of ReLu function is

(4) $$\begin{array}{c} f\left(x\right)=\text{max}\left(0,x\right). \end{array}$$

The full name of ReLU function is rectified linear unit. The function is one of the commonly used activation functions, which are characterized by low-computational complexity and no exponential operation. However, it is worth explaining that ReLU function has certain defects in the calculation process. When the data passes through the negative range of ReLU function, the output value is equal to 0. The Leaky–ReLu function can solve the above problem.

(5) $$\begin{array}{c} f\left(x\right)=\left\{\begin{array}{c} x,x\geq 0,\\ \alpha x,x<0. \end{array}\right. \end{array}$$

Therefore, the efficiency of the entire network operation can be improved to a certain extent. The corresponding equations of Sig and Tanh are as follows:

(6) $$\begin{array}{c} \left\{\begin{array}{c} \text{tanh}\left(x\right)=\frac{\text{exp}\left(x\right)-\text{exp}\left(-x\right)}{\text{exp}\left(x\right)+\text{exp}\left(-x\right)}, \end{array}\right. \end{array}$$

(7) $$\begin{array}{c} h_{w,b}\left(x_{i}\right)=\left[\begin{array}{c} p\left(y_{i}=2|x_{i};w,b\right)\\ p\left(y_{i}=3|x_{i};w,b\right)\\ \cdots \\ p\left(y_{i}=n|x_{i};w,b\right) \end{array}\right] \end{array}$$

The output layer adopts softmax function to normalize, and the probability value in the corresponding category is shown in equation (7). In the classification tasks, *i* is the cross entropy (CE) loss function that is often used to evaluate the gap between predicted value and true value. The CE formula is as follows:

(8) $$\begin{array}{c} \text{loss}=-\frac{1}{m}\sum_{j=1}^{m}\sum_{i=1}^{n}y_{ji}\log\left({\hat{y}}_{ji}\right), \end{array}$$

where ${\hat{y}}_{ji}$ is the predicted value and *y*_*ji*_ is the real value. The error calculated from the CE function needs to be calculated by back propagation, so as to realize the newer back propagation of model parameters. The original form of the gradient descent method is shown in equation (9):

(9) $$\begin{array}{c} \theta ≔=\theta -\alpha \frac{\partial }{\partial \theta }J\left(\theta \right). \end{array}$$

In the experiments in the following sections, this paper also verifies that the use of Adam has faster convergence than SGD. The mathematical expression of a common Adam optimizer is given as follows:

(10) $$\begin{array}{c} m_{t}=\beta_{1}m_{t-1}+\left(1-\beta_{1}\right)g_{t},\\ v_{t}=\beta_{2}v_{t-1}+\left(1-\beta_{2}\right)g_{\text{t}}^{2}. \end{array}$$

Therefore, the updating rule of gradient descent is as follows:

(11) $$\begin{array}{c} \theta_{t+1}=\theta_{t}-\frac{\alpha }{\sqrt{v_{t}+\epsilon }}m_{t}. \end{array}$$

### 3.2. Optimized CNN Model

It is worth noting that differential perturbation is used in this paper to optimize the CNN model, but other optimization algorithms are feasible in this theory, but they are not optimal choices. Particle swarm optimization (PSO) is simple and easy to solve, but it is prone to local extreme points, low accuracy, slow convergence, and stagnation. In this section, the differential perturbation is introduced into the PSO to form the differential perturbation particle swarm optimization (DPPSO) algorithm, which makes use of the advantages of fast convergence speed and good global performance of difference, overcomes the shortcomings of low precision and local optimal caused by PSO, and builds an optimized CNN model. The multiobjective optimization model is

(12) $$\begin{array}{c} \text{min}f_{1}\left(x_{1},x_{2}\right),\\ \text{max}f_{2}\left(x_{1},x_{2}\right). \end{array}$$

s.t.

(13) $$\begin{array}{c} p_{1}<g_{1}\left(x_{1},x_{2}\right)<q_{1},\\ p_{2}<g_{2}\left(x_{1},x_{2}\right)<q_{2},\\ p_{3}<g_{3}\left(x_{1},x_{2}\right)<q_{3},\\ p_{4}<g_{4}\left(x_{1},x_{2}\right)<q_{4}. \end{array}$$

and

(14) $$\begin{array}{c} 120<x_{1}<180,\\ 120<x_{2}<180, \end{array}$$

where *f*_1_ represents energy consumption target, *f*_2_ represents the output target g_{1}, g_{2}, g_{3}, g_{4}*g*_1_(*x*_1_, *x*_2_), *g*_2_(*x*_1_, *x*_2_), *g*_3_(*x*_1_, *x*_2_), *g*_4_(*x*_1_, *x*_2_) represent the packaging quality of four indicators: crushing strength, wear strength, drop strength, compressive strength, respectively. It is worth noting that the DPPSO algorithm used in this paper optimizes network parameters to obtain better model performance.

**Figure 2:**
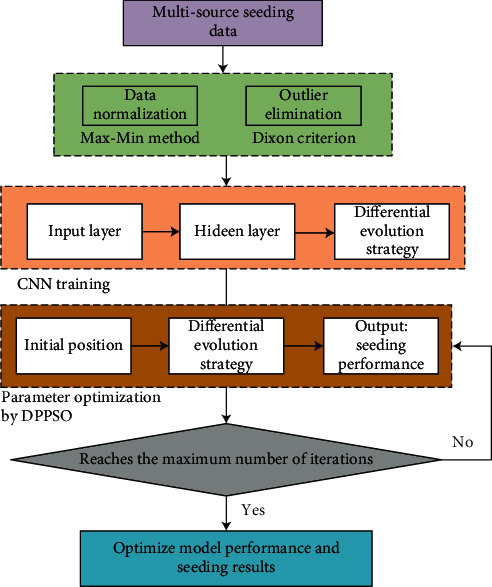
The framework of the proposed method in this paper.

Based on the above discussions, the optimized deep neural network and its application in fine sowing of crops is shown in Figure 2. It mainly includes data preprocessing, CNN model training, and parameter optimization based on DPPSO model, and finally obtains the optimal model performance.

## 4. Experimental Results and Analysis

### 4.1. Experimental Data Introduction

This area belonged to semiarid and semihumid winter wheat growing. The experimental site was a hilly dry land with an average annual rainfall of about 450 mm. The test field was flat and has one cropping system in a year, and the soil was medium alkaline clay loam. The water storage in the test area was mainly natural precipitation, which was concentrated in October and November of 2019. The experimental variety Linfeng no. 3 was provided by the County Agricultural Committee. The experiment used a two-factor experimental design. Furrow sowing (FS) was the main sowing area, and furrow sowing (FS), wide drilling sowing (WDS), and conventional drilling sowing (CDS) were the main sowing methods.

In addition, this study referred to the following data sources: China Rural Statistical Yearbook (1998–2019), China Statistical Yearbook (19982020), and National Agricultural Product Cost–Benefit Data Collection (1998–2020). Excel 2019 software and DPS7.05 software were used for statistical collation of data, Excel 2019 software was used for plotting, and least significant difference (LSD) method was used for significance test of difference, reaching significance level *a* = 0.05.

### 4.2. Experimental Results Analysis

**Figure 3:**
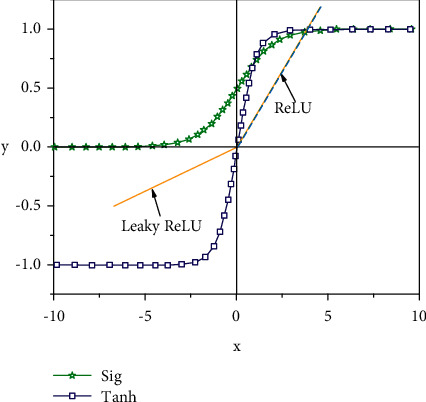
Different activation functions curves of CNN model.

In order to demonstrate the universality of the proposed method, change curves of different activation functions of CNN model are presented in Figure 3. They all have the following common characteristics: (1) differentiability: this property is a prerequisite when using gradient-based optimization algorithms to optimize models. (2) Monotonicity: when the activation function meets the monotonicity, the single-layer network is guaranteed to be convex so that the subsequent convex optimization operations can be carried out. But in this case, the learning rate usually needs to be set to a small value, which inevitably increases the training time.

**Figure 4:**
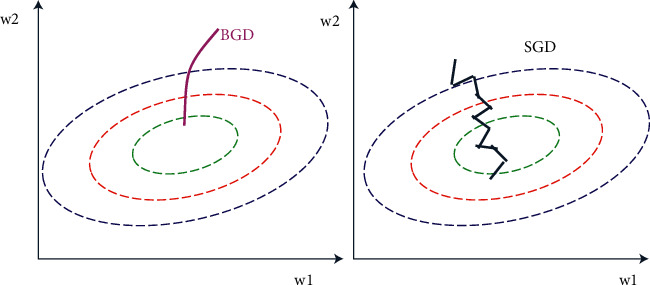
BGD vs. SGD.

In order to verify the training performance of the model, different parameter updating methods are presented, as shown in Figure 4. The left is the batch gradient descent (BGD) algorithm, which refers to the calculation error every time, the gradient is obtained by the same batch as a whole, and the parameters are constantly updated until the error is zero or within the allowed range. The right is the stochastic gradient descent (SGD) algorithm, which means that the training of each sample is updated once, and the data order needs to be shuffled before each cycle. From Figure 4, we know that a big problem of BGD is that the whole data set needs to be scanned in each iteration of gradient calculation. Therefore, when the data volume is large, it inevitably leads to a large amount of calculation and low efficiency, while SGD only needs to take one sample point in each iteration of gradient calculation, so it has computational advantages. Second, since the gradient calculated by SGD is very different from the real negative gradient, it is not very stable, which also explains one of the advantages of SGD, which can jump out of the local optimal solution, so as to find the real global optimal solution. This is especially important in deep learning, where objective functions tend to be nonconvex. In conclusion, SGD model not only runs faster than BGD model in training time. In addition, SGD model solves the problem that BGD model can easily fall into local optimum. Hence, the SGD is used to update the model parameters in this paper.

**Figure 5:**
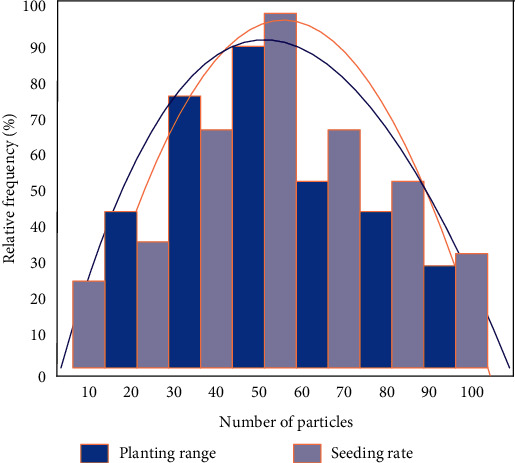
Distribution of sowing quantity and sowing area.

In order to verify the effective control of the proposed method on wheat sowing range and sowing quantity, according to the determination method of seeding uniformity recorded in national standard GB/T 9478–2005, after the seeding operation is completed, a total of 30 sections of 10 cm were taken, and the number of seed particles in each section was counted, as shown in Figure 5. Sowing uniformity was calculated for each level of combination seeding operation. It can be seen from the figure that the relative frequency distribution of wheat grain number in each subsection presents positive distribution. The experimental results were analyzed uniformly under the same theoretical sowing rate per hectare. Specifically, when the number of seeds is 50–60, both the sowing range and the sowing rate are the highest, so the result can be considered as the optimal sowing amount per unit area.

**Figure 6:**
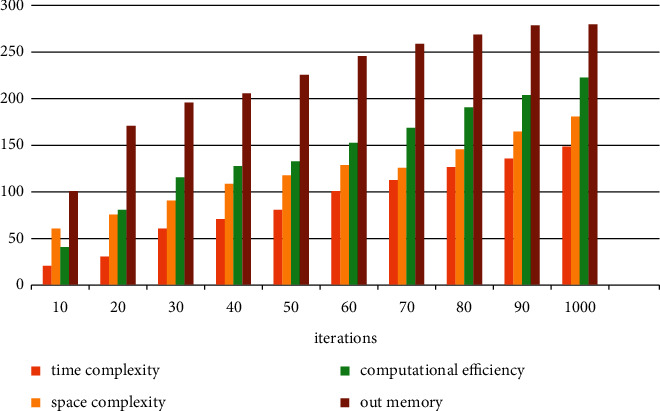
The computational efficiency and actual complexity of the proposed method.

Figure 6 shows the computational efficiency and actual complexity of the proposed method. As can be seen from the figure, the computational complexity and efficiency of the proposed method increase first and then decrease with the increase of iterations. In other words, the computational efficiency of the method in this paper reaches the maximum after iteration at 1000 hours. Therefore, the proposed method has a large model fault tolerance rate, which can ensure good model performance within 1000 iterations. It also indirectly shows that the method proposed in this paper has good generalization and extensibility.

**Figure 7:**
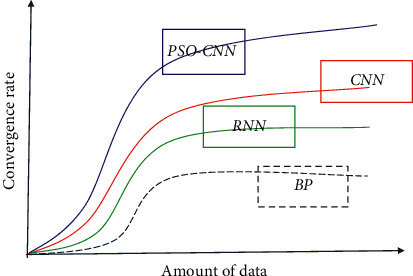
Comparison of convergence speed between different methods.

Because BP neural network is a shallow model, RNN is a classic deep learning model, and the method in this paper is based on CNN model. Hence, they are selected as the comparison algorithms and the simulation results are presented in Figure 7. As can be seen from the figure, the convergence rates of the four methods all were tended to increase first with the increase of data volume, but with the further increase of data volume, the convergence rate of BP neural network showed a downward trend, indicating that BP neural network is not suitable for processing a large amount of wheat sowing data in this paper. In contrast, the convergence rate of RNN and CNN models generally keeps increasing with the increase of data volume at any time. The main reason is that both methods are deep neural networks with the ability to process big data. However, the convergence rate of these two methods is still not as high as that of PSO-CNN in this paper, which shows the effectiveness and practicability of the method proposed in this paper.

**Figure 8:**
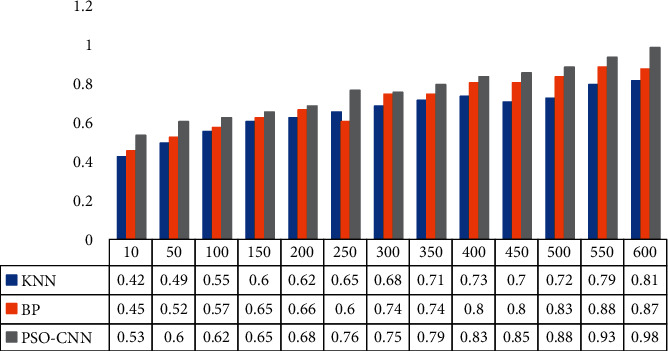
Comparison of sowing accuracy of different methods.

Figure 8 shows the relationship between sowing accuracy and iteration times of different methods. We can figure out from the figure whether with the increase of iteration number, the sowing accuracy of the three methods shows an increasing trend. When the iteration number is 600, the three models all reach the highest classification accuracy, which is 81%, 87%, and 98%, respectively. Therefore, the PSO-CNN model in this paper achieves the highest classification accuracy. In addition, even when the number of iterations is small, the proposed method also has the best model performance and achieves the highest classification accuracy throughout the training process, which demonstrates the effectiveness of the proposed method in wheat seeding monitoring.

**Figure 9:**
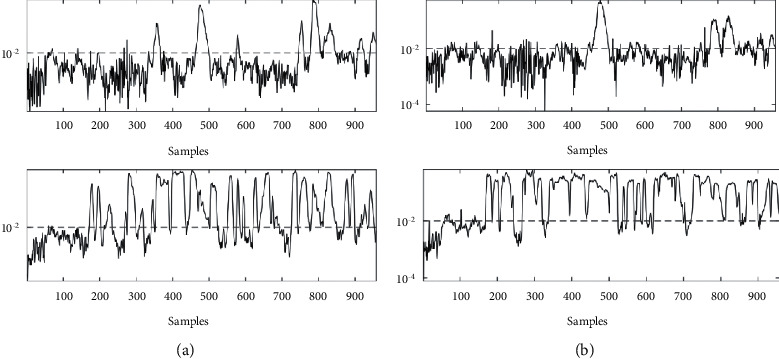
Monitoring accuracy of wheat seeding by (a) CNN, (b) PSO-CNN.

To better demonstrate the effectiveness of the proposed method, the monitoring results of CNN and PSO-CNN are shown in Figure 9. Specifically, it can be seen from Figure 9(b) that PSO-CNN method not only achieved the lowest omission ratio of 18.88% but also detected abnormal sowing at the 163rd sampling point with a detection delay of 2, while the corresponding delay numbers of CNN method were 24, respectively. It shows that the method presented in this paper can detect the sowing error quickly. In addition, when the error is detected, the statistical curve corresponding to the PSO-CNN model rarely falls below the threshold line, while the statistical curve corresponding to the CNN method always falls back to different degrees, resulting in a high failure rate, which further demonstrates the stability and persistence of the proposed method.

## 5. Conclusions

Sowing is a key link in wheat production. The performance of seeding machine directly affects the growth and yield of crops. With the promotion of precision agriculture and the development of precision seeding technology, precision seeding has become the main component of modern agricultural seeding technology system. Adopting precision seeding technology is an important means of large-scale production and realizing cost-saving and efficiency enhancement. The online precision measurement of seeding amount is the key to realize precision seeding and precise control and also the basis of realizing precision seeding in real sense.

In view of the shortcomings of the existing methods, this paper proposed an optimized deep learning model PSO-CNN, which not only achieved better model convergence rate and model parameters but also effectively improved the sowing accuracy and sowing range of wheat showing strong theoretical value and application potential. This work is helpful to realize the fine sowing of wheat and improve the level of agricultural automation. Although the method proposed in this paper has achieved good results, the research in this paper does not consider the effects of planting weather and soil in the process of agricultural sowing. This will be the focus of future research.

## Data Availability

The experimental data used to support the findings of this study are available from the corresponding author upon request.

## References

[1] Cui K., Shoemaker S. P. (2018). Public perception of genetically-modified (gm) food: a nationwide Chinese consumer study. *Npj Science of Food*.

[2] Huang G. Q., Tsai F. S. (2021). Social innovation for food security and tourism poverty alleviation: some examples from China. *Frontiers in Psychology*.

[3] Yu J., Han Q. (2020). Food security of mariculture in China: e. *Marine Policy*.

[4] Chen L., Chang J., Wang Y. (2021). Disclosing the future food security risk of China based on crop production and water scarcity under diverse socioeconomic and climate scenarios. *Science of the Total Environment*.

[5] Pu M., Zhong Y. (2020). Rising concerns over agricultural production as COVID-19 spreads: l. *Global Food Security*.

[6] Gao H., Yan C., Liu Q., Ding W., Chen B., Li Z. (2019). Effects of plastic mulching and plastic residue on agricultural production: a meta-analysis. *Science of the Total Environment*.

[7] Maresma A., Ballesta A., Santiveri F., Loveras J. (2019). Sowing date affects maize development and yield in irrigated mediterranean environments. *Agriculture*.

[8] Mack L., Munz S., Capezzone F. (2018). Sowing date in Egypt affects chia seed yield and quality. *Agronomy Journal*.

[9] Švamberková E., Doležal J., Lepš J. (2019). The legacy of initial sowing after 20 years of ex-arable land colonisation. *Oecologia*.

[10] Herman D. I., Weerasekara C., Hutcherson L. C. (2021). Precise multispecies agricultural gas flux determined using broadband open-path dual-comb spectroscopy. *Science Advances*.

[11] Yan Z., Jingtao H. (2019). The precise positioning algorithm optimization base on PSO-PF for agricultural machinery navigation system Journal of physics: conference series. *Journal of Physics: Conference Series*.

[12] Lin X. Z., Lin C. X., Wang X., Xue J., Zheng E. (2018). Precise positioning of agricultural vehicles under static conditions via artificial intelligence algorithms. *International Agricultural Engineering Journal*.

[13] Coccia M. (2020). Deep learning technology for improving cancer care in society: new directions in cancer imaging driven by artificial intelligence. *Technology in Society*.

[14] Yamada M., Saito Y., Imaoka H. (2019). Development of a real-time endoscopic image diagnosis support system using deep learning technology in colonoscopy. *Scientific Reports*.

[15] Verma S., Kumar N., Verma A., Singh H., Siddique K. H. M., Singh N. P. (2020). Novel approaches to mitigate heat stress impacts on crop growth and development. *Plant Physiology Reports*.

[16] Feng F., Li Y., Qin X., Liao Y., Siddique K. H. M. (2017). Changes in rice grain quality of indica and japonica type varieties released in China from 2000 to 2014. *Frontiers of Plant Science*.

[17] Ozturk A., Erdem E., Aydin M., Karaoglu M. M. (2022). The effects of drought after anthesis on the grain quality of bread wheat depend on drought severity and drought resistance of the variety. *Cereal Research Communications*.

[18] Liu Q., Ma H., Lin X., Zhou X., Zhao Q. (2019). Effects of different types of fertilizers application on rice grain quality. *Chilean Journal of Agricultural Research*.

[19] Dong Z., Zhang X., Li J. (2019). Photosynthetic characteristics and grain yield of winter wheat (Triticum aestivum L.) in response to fertilizer, precipitation, and soil water storage before sowing under the ridge and furrow system: a path analysis. *Agricultural and Forest Meteorology*.

[20] Wang H., Ke F., Wang H., Zhao Y. (2019). Effects of sowing time, density and fertilizer application on the economic characters of Zhuoyou 058. *Agricultural Biotechnology*.

[21] Gierz Ł, Kruszelnicka W., Robakowska M. (2022). Optimization of the sowing unit of a piezoelectrical sensor chamber with the use of grain motion modeling by means of the discrete element method. Case study: rape seed. *Applied Sciences*.

[22] Matsera O. (2020). Comparative evaluation of quality properties of winter rapeseed depending on the level of fertilizers and sowing date. *Agriculture and Forestry*.

[23] Romaneckas K., Steponavičius D., Jasinskas A. (2022). How to analyze, detect and adjust variable seedbed depth in site-specific sowing systems: a case study. *Agronomy*.

[24] Muscalu A., Tudora C., Vlăduțoiu L., Vlad C., Dorogan A. Precision sowing of vegetable seeds using electrically operated distribution devices[C]//E3S Web of Conferences.

[25] Onakpa M. M., Njan A. A., Kalu O. C. (2018). A review of heavy metal contamination of food crops in Nigeria. *Annals of global health*.

[26] Savary S., Willocquet L., Pethybridge S. J., Esker P., McRoberts N., Nelson A. (2019). The global burden of pathogens and pests on major food crops. *Nature ecology & evolution*.

[27] Gao X., Cui T., Zhou Z. (2021). DEM study of particle motion in novel high-speed seed metering device. *Advanced Powder Technology*.

[28] Ibrahim E. J., Elfadil A. D., Abdallah A. D. (2022). Laboratory and field investigation comparison for seed distribution accuracy of a multi-rows pneumatic plate metering device. *Asian Journal of Agriculture and Food Sciences*.

[29] Kumar A., Vashishtha G., Gandhi C. P., Zhou Y., Glowacz A., Xiang J. (2021). Novel convolutional neural network (NCNN) for the diagnosis of bearing defects in rotary machinery. *IEEE Transactions on Instrumentation and Measurement*.

[30] Wang Z., Zhao W., Du W., Li N., Wang J. (2021). Data-driven fault diagnosis method based on the conversion of erosion operation signals into images and convolutional neural network. *Process Safety and Environmental Protection*.

